# Vocal emotion perception in schizophrenia and its diagnostic significance

**DOI:** 10.1186/s12888-023-05110-2

**Published:** 2023-10-17

**Authors:** Wenxuan Zhao, Qi Zhang, Huimei An, Yajun Yun, Ning Fan, Shaoxiao Yan, Mingyuan Gan, Shuping Tan, Fude Yang

**Affiliations:** 1 Beijing HuiLongGuan Hospital, Peking University HuiLongGuan Clinical Medical School, No 7, HuangtuNandian, ChangPing District, Beijing, 100096 China; 2 Wuxi Mental Health Center, Wuxi, China

**Keywords:** Schizophrenia, Vocal emotion perception, Cognition

## Abstract

**Background:**

Cognitive and emotional impairment are among the core features of schizophrenia; assessment of vocal emotion recognition may facilitate the detection of schizophrenia. We explored the differences between cognitive and social aspects of emotion using vocal emotion recognition and detailed clinical characterization.

**Methods:**

Clinical symptoms and social and cognitive functioning were assessed by trained clinical psychiatrists. A vocal emotion perception test, including an assessment of emotion recognition and emotional intensity, was conducted. One-hundred-six patients with schizophrenia (SCZ) and 230 healthy controls (HCs) were recruited.

**Results:**

Considering emotion recognition, scores for all emotion categories were significantly lower in SCZ compared to HC. Considering emotional intensity, scores for anger, calmness, sadness, and surprise were significantly lower in the SCZs. Vocal recognition patterns showed a trend of unification and simplification in SCZs. A direct correlation was confirmed between vocal recognition impairment and cognition. In diagnostic tests, only the total score of vocal emotion recognition was a reliable index for the presence of schizophrenia.

**Conclusions:**

This study shows that patients with schizophrenia are characterized by impaired vocal emotion perception. Furthermore, explicit and implicit vocal emotion perception processing in individuals with schizophrenia are viewed as distinct entities. This study provides a voice recognition tool to facilitate and improve the diagnosis of schizophrenia.

**Supplementary Information:**

The online version contains supplementary material available at 10.1186/s12888-023-05110-2.

## Background

Schizophrenia is a chronic psychiatric disorder that strongly interferes with major areas of life, including education, work, and daily living. More than 20 million people worldwide suffer from schizophrenia, with a higher rate in men compared to women [1]. Patients with schizophrenia are characterized by serious impairments in social cognition [2, 3], including distortion of emotion and language and social isolation, also resulting in difficulties communicating [4]. Emotional cognitive impairment is another key feature of schizophrenia [5]. To date, many studies have found that patients with schizophrenia show reduced emotional expression, impaired vocal emotion recognition, and impaired understanding of emotional expressions [6]. Vocal emotion recognition plays a crucial role in social communication and is a key element in the detection of schizophrenia [7, 8].

Studies on patients with schizophrenia have found a significant correlation between vocal emotion recognition, cognition, and clinical factors (such as difficulties in auditory processing, the severity of the disease, and negative symptoms [9]). Research on cognition also suggested that dysfunction in vocal emotion recognition in patients with schizophrenia occurs before the apparent disease onset [10–12]. However, findings in this regard have been controversial, due to differences in study design and presented stimuli, sex distribution in the sample as well as language background (language type and structure), and differing positive symptoms of schizophrenia [9, 13, 14]. These findings are of great significance to clinical practice and research, as they might highlight the need to develop new and alternative methods to identify and treat patients with schizophrenia and social functioning deficiencies due to mental disorders in general.

Research on vocal emotion recognition can be extended to other neurocognitive fields such as memory, monitoring, thinking and reasoning, literacy, language production, and problem-solving ability. Therefore, in our study, a new voice recognition method was used to systematically explore differences between cognitive and social aspects of emotions as observed in voice emotion recognition, based on epidemiological data, clinical manifestations, and cognitive function screening. Moreover, to determine their functions and clinical significance, we attempted to clarify the effectiveness of this tool in assessing the ability to recognize vocal emotions.

## Materials and methods

### Participants

Participants were recruited from Beijing Huilongguan Hospital from August 2020 to May 2022. All participants provided written informed consent before undergoing any research procedure. The study protocol was conducted in accordance with the Declaration of Helsinki and was approved by the research ethics and institutional review boards of Beijing Huilongguan Hospital.

Inclusion criteria for the patient group were as follows: (1) the patient met the diagnostic criteria of schizophrenia in the Diagnostic and Statistical Manual of Mental Disorders 5th Edition (DSM-V); (3) the patient was between 18 and 60 years of age and had completed more than six years of education; (4) patients and their family members voluntarily participated in the study and signed the informed consent form; (5) the patient’s condition was stable and he/she was able to communicate effectively. The exclusion criteria were as follows: (1) intellectual disability or any brain organic disease; (2) severe recession or impulsive excitement and uncooperative behavior; (3) severe depression, anxiety, and substance abuse; and (4) serious physical disease or drug side effects, which made communication impossible. In total, 106 patients with schizophrenia were enrolled.

The criteria for the healthy control group (230 participants) were as follows: (1) 18–60 years of age; (2) education level of junior high school or above; (3) fluency in Mandarin; (4) clear articulation, and no articulation disorder; (5) no family history of mental illness; (6) healthy mental state, no evidence of anxiety and depression (as attested by a psychiatric interview); (7) the scores of the Self-rating Anxiety Scale (SAS; Dunstan and Scott [15]) and Self-rating Depression Scale (SDS; Dunstan et al. [16]) in the normal range (< 30 points); and (8) normal (or normal after correction) hearing.

### Neuropsychological and psychopathological assessment

The evaluation of scales was performed by a team of trained psychiatrists using the routine examination method, and there was good consistency among the measures (intraclass correlation coefficient, ICC > 0.8). The methods are detailed further in the Supplementary Methods.

### Basic information questionnaire

A self-assessed general information questionnaire was used to collect data on sex, age, years of education, and course of the disease.

### Clinical symptom and social function assessment

The Temporary Experience of Pleasure Scale (TEPS; Chan et al. [17]), Personal and Social Performance Scale (PSP [18]), Positive and Negative Symptom Scale (PANSS; Kirkpatrick et al. [19]), and Brief Negative Symptom Scale (BNSS; Kirkpatrick et al. [19]) were used to evaluate psychotic symptoms in the schizophrenia group.

### Cognitive function

The Chinese version of the Consensus Cognitive Battery, originally developed by the National Institute of Mental Health (NIMH) Measurement and Treatment Research to Improve Cognition in Schizophrenia (MATRICS) initiative (MCCB [20]), was used to assess cognitive functioning. It includes seven aspects: speed of processing, working memory, verbal learning and memory, visual learning and memory, reasoning and problem-solving, attention or vigilance, and social cognition. There are ten sub-tests.

### Vocal emotion perception evaluation

The vocal emotion recognition task comprised 42 standardized emotional voices, performed by two professional sex-specific drama actors, including seven emotions: anger, calmness, disgust, fear, sadness, irony, and surprise. There were six voices for each emotion, including three male and three female voices. High and low materials were also manipulated, with different emotional intensities. The intensity was graded on a 100-point scale. The higher the score, the greater the emotional intensity. After completing the phonetic emotion judgment, the participants entered the next trial, and all trials were presented in a pseudo-randomized order. The evaluation indicators included the emotional category score (the number of attempts to correctly identify a certain phonetic emotional category, with a maximum score of six points) and emotional intensity score (the corresponding emotional intensity after correctly identifying a certain emotional category, with a maximum score of 100 points). See the Supplementary Methods for further details.

## Results

### Demographics, clinical characteristics, the cognitive function of the cohort

Finally, 106 patients of schizophrenia (SCZ) and 230 healthy controls (HCs) were recruited in the study. We observed no significant difference in the basic demographic data between the patient and control groups. With regard to the severity of schizophrenia, the total PANSS score was 59.39 ± 20.54, that of BNSS was 1.86 ± 1.98, and PSP was 69.38 ± 13.54. The course of disease was 11.22 ± 8.86 years. The total scores of TEPS and MCCB of the patients were significantly lower than those of the healthy controls (*p* < 0.01). Further details are provided in Table 1. Baseline data suggested that in the cohort, patients and healthy controls were matched in age, sex, and years of education, while the evalution of the patients in scales (TEPS and MCCB) was consistent with the previous studies [19, 20], with statistically significant differences, which indicated vocal emotion perception tests could be further applied to further correlation research to explain the disease characteristics.

**Table 1:** General demographic data of patients with schizophrenia and healthy controls

Project		SCZ (n = 106)	HCs (n = 230)	*p*-value
Sex (male/female)		52/54	102/128	0.448
Age (years)		34.83 ± 9.91	35.69 ± 11.20	0.507
Years of education (years)		14.13 ± 3.068	14.42 ± 3.03	0.519
TEPS total score		77.88 ± 2.97	62.51 ± 0.28	< 0.001
MCCB total score		39.78 ± 39.78	55.29 ± 7.51	< 0.001
PANSS	Positive symptom score	14.11 ± 6.37	-	-
	Negative symptom score	15.34 ± 6.07	-	-
	General psychopathology score	29.94 ± 10.21	-	-
	PANSS total score	59.39 ± 20.54	-	-
BNSS	Anhedonia score	3.08 ± 3.27	-	-
	Distress score	1.15 ± 1.29	-	-
	Asociality score	2.77 ± 2.40	-	-
	Avolition score	2.47 ± 2.34	-	-
	Blunted affect Score	3.40 ± 3.49	-	-
	Alogia score	2.03 ± 1.98		
	BNSS total score	14.50 ± 7.32	-	-
PSP	PSP total score	69.38 ± 13.54	-	-
Drug dosage	Chlorpromazine equivalents(mg)	595.99 ± 314.57	-	-
Disease course	Disease course (years)	11.22 ± 8.86	-	-

Note. SCZ, schizophrenia; HCs, healthy controls; TEPS, Temporal Experience of Pleasure Scale; MCCB, Measurement and Treatment Research to Improve Cognition in Schizophrenia (MATRICS) Consensus Cognitive Battery; PANSS, Positive and Negative Symptom Scale; BNSS, Brief Negative Symptom Scale; PSP, Personal and Social Performance Scale; chlorpromazine equivalents convert the drugs currently used by patients into chlorpromazine equivalents according to the drug dosage conversion standard. The highest equivalent of chlorpromazine in the schizophrenia group was 2,400 mg/day and the lowest equivalent was 75 mg/day

### Comparison of vocal emotion recognition and intensity scores between patients with schizophrenia (SCZ) and healthy control (HC) groups

#### Vocal emotion recognition and intensity

Patients’ total scores and their scores for all categories of emotions were significantly lower than those of healthy controls (*p* < 0.001). However, while the patients’ overall scores for recognition of vocal emotion intensity were still lower than those of healthy controls, only the patients’ scores for anger, calmness, sadness, and surprise were significantly lower than those of healthy controls (*p* < 0.001). Further details are provided in Table 2.

**Fig. 1:**
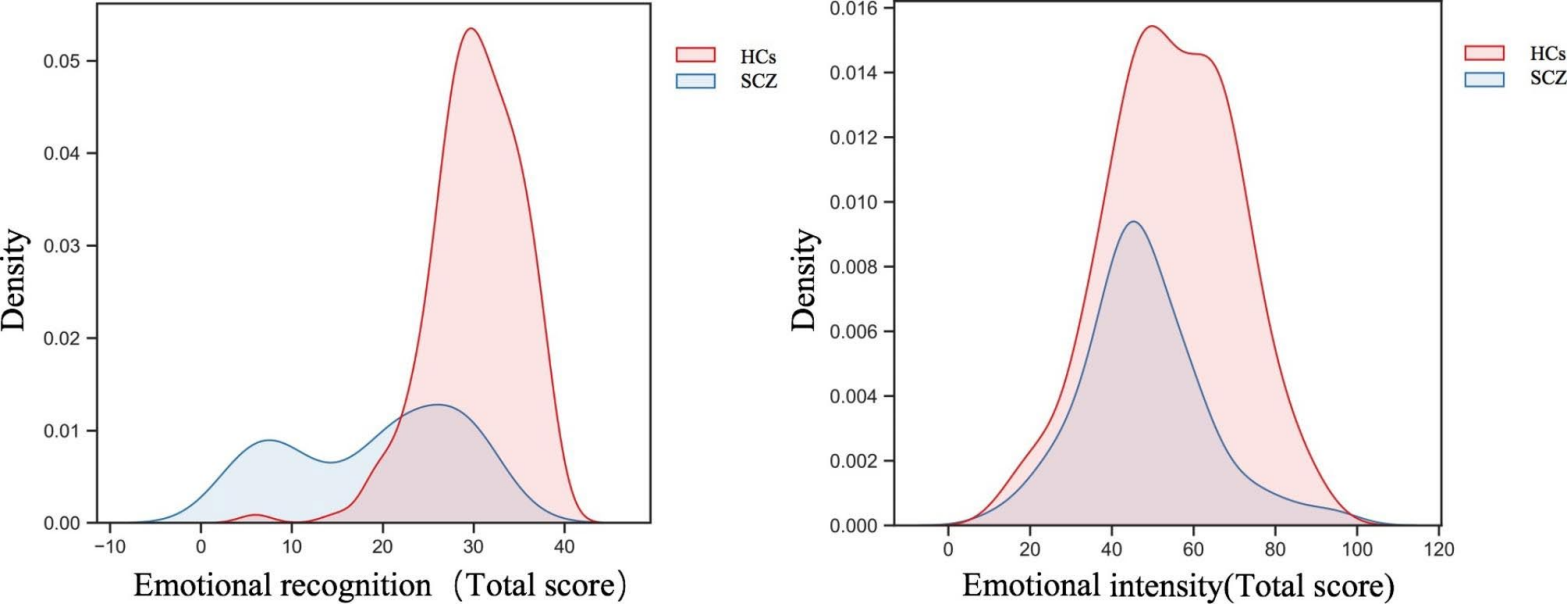
Density plots of emotion recognition and intensity: the density plots indicated a different distribution of emotional recognition and intensity scores (HCs, healthy controls; SCZ, schizophrenia)

Additionally, we found significant differences in vocal emotion recognition and intensity recognition models between the SCZ and HC groups. The total score for emotional intensity and recognition was significantly correlated with detailed classifications (including anger, calmness, disgust, fear, sadness, satire, and surprise; Pearson’s correlation coefficient *r* > 0.9, *p* < 0.001). Differences can be observed in density plots for recognition and intensity (Fig. 1).

**Fig. 2:**
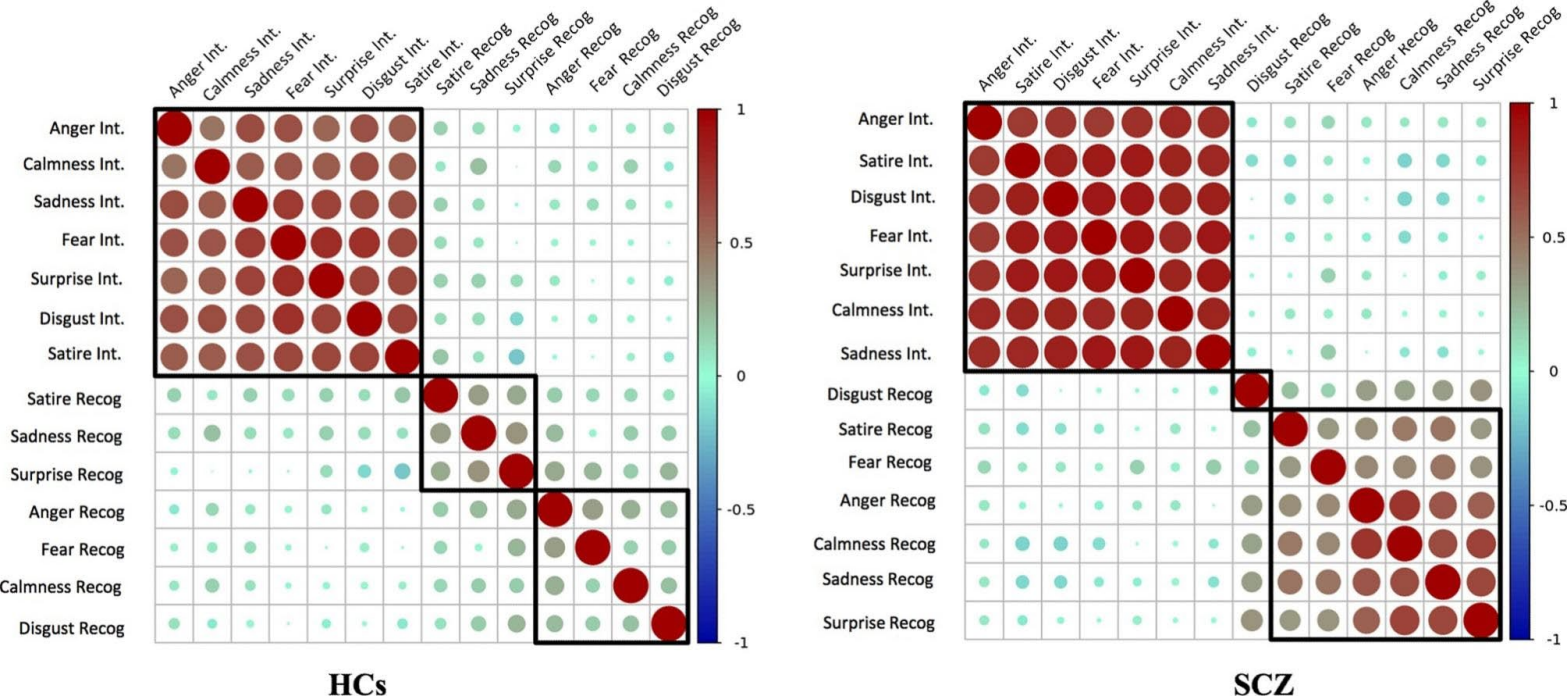
Correlation between vocal emotion recognition sub-scales. Consistency across recognition and intensity is not the same between patients and controls. Pairwise Pearson correlation for recognition has increased in patients (*r* > 0.8, *p* < 0.001) while the pattern was similar for emotional intensity. (HCs, healthy controls; SCZ, schizophrenia; Int., intensity; Recog., recognition, the legend represents the correlation coefficient, red represents positive correlation, and blue represents negative correlation; circle size represents significance; black boxes represent cluster analysis (Pearson correlation, *r* > 0.8)

Moreover, using a correlation coefficient graph (Fig. 2 [21]), we tried to verify whether the pattern for recognition and intensity was the same between SCZ and HCs. Consistency across recognition differed between SCZ and HCs. The pairwise Pearson’s correlation of the recognition scores for patients increased (*r* > 0.8, *p* < 0.001), while the pattern was similar for emotional intensity [21].

**Table 2:** Comparison of emotion recognition and intensity between SCZ and HCs group

Emotion	Subject	SCZ	HCs	*p*-value
Anger	Recognition	3.52 ± 1.88	4.98 ± 1.18	< 0.001
	Intensity	52.58 ± 17.17	69.90 ± 14.87	< 0.001
Calmness	Recognition	3.85 ± 2.16	5.66 ± 0.69	< 0.001
	Intensity	48.07 ± 14.84	57.48 ± 22.26	< 0.001
Disgust	Recognition	1.73 ± 1.38	3.47 ± 1.58	< 0.001
	Intensity	47.61 ± 16.75	49.39 ± 19.40	0.432
Fear	Recognition	2.37 ± 1.81	4.14 ± 1.67	< 0.001
	Intensity	46.31 ± 16.26	48.52 ± 19.80	0.322
Sadness	Recognition	3.04 ± 1.81	4.40 ± 1.43	< 0.001
	Intensity	47.19 ± 16.77	57.27 ± 18.01	< 0.001
Satire	Recognition	1.89 ± 1.59	3.07 ± 1.65	< 0.001
	Intensity	46.47 ± 16.17	46.78 ± 20.32	0.892
Surprise	Recognition	2.72 ± 2.09	4.06 ± 1.59	< 0.001
	Intensity	46.85 ± 16.50	52.31 ± 18.80	0.014
Total score	Recognition	19.2 ± 9.04	30.19 ± 4.99	< 0.001
	Intensity	47.87 ± 15.08	54.67 ± 15.88	< 0.001

SCZ, schizophrenia; HCs, healthy controls

#### Correlation between vocal emotion recognition and clinical features

In the current study, we did not identify any significant correlation between sex, age, the clinical course of the disease, and drug dosage (chlorpromazine equivalent (mg)) with vocal emotion recognition and intensity discrimination(*p* > 0.1). Correlation analysis with PANSS (including 4 subscales) and BNSS (including 7 subscales) showed no significance(*p* > 0.05).

#### Correlation between vocal emotion recognition and cognitive function

After multiple regression analysis, we found that the content of vocal emotion recognition was significantly related to cognitive function in patients (ANOVA, F = 3.61, *p* = 0.002), and, specifically, to working memory (*p* = 0.015, Table 3); however, vocal emotional intensity was not related to cognitive function (ANOVA, F = 1.51, *p* = 0.32).

**Table 3:** Multiple regression analysis between vocal emotion recognition and cognitive function

Project	Standardized coefficients (Beta)	*t*	*p*-value
Verbal learning	0.19	1.22	0.23
Reasoning and problem-solving	0.042	0.30	0.76
Visual learning	-0.23	-1.44	0.16
Social cognition	0.027	0.21	0.84
Attention/vigilance	0.20	1.24	0.22
Speed of processing	0.12	0.85	0.40
Working memory	0.37	2.53	0.015

The vocal recognition content did not correlate with TEPS (ANOVA, F = 0.45, *p* = 0.77) nor PSP (ANOVA, F = 0.17, *p* = 0.68). The vocal emotional intensity was not correlated with TEPS (ANOVA, F = 1.15, *p* = 0.34) nor PSP (ANOVA, F = 0.76, *p* = 0.39).

### Emotion recognition assisted clinical discrimination results

**Fig. 3:**
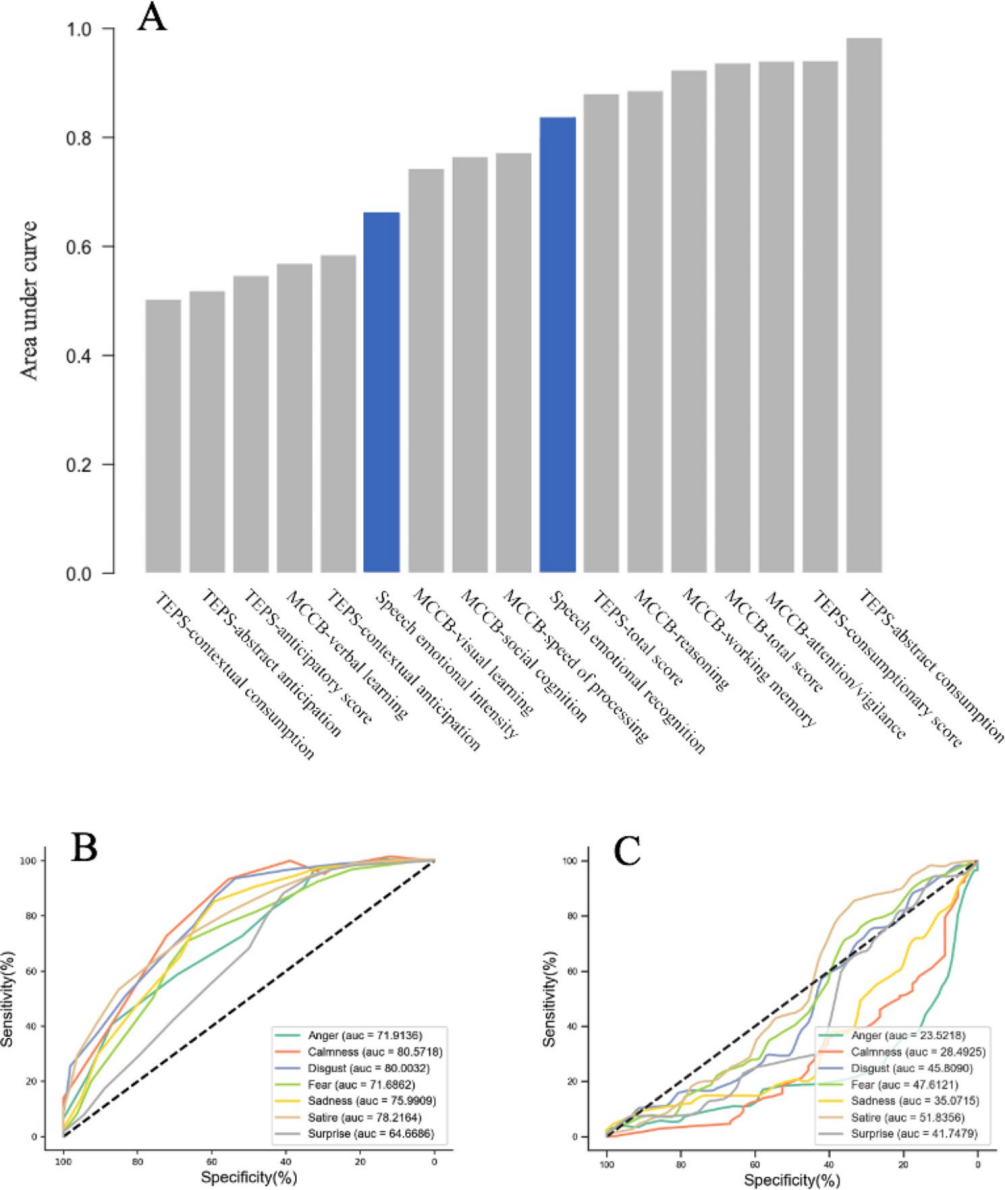
The clinical receiver operating characteristic curve (ROC) analysis. Figure A compares the ROC analysis of cognitive function tools (MCCB tools), clinical emotion recognition tools (TEPS), and presented vocal recognition tools in the diagnosis of schizophrenia in this study, in which voice emotion recognition indicates a relatively satisfied area under the curve (AUC: 84%, *p* < 0.01), and sound intensity efficiency is relatively poor (62%). The analysis of detailed emotions is shown in Fig. 3B-C, Figure B shows the ROC analysis of vocal recognition, and Figure C shows the analysis of intensity (AUC).

Based on the phenotypes of patients and controls, we conducted diagnostic tests to determine the sensitivity and specificity of clinical indicators. The results showed that the total score of vocal emotion recognition was a good indicator of the presence of schizophrenia (AUC = 84%, *p* < 0.01), but the respective intensity was not an ideal indicator (AUC < 50%). The overall evaluation efficiency was lower than the total score of the MCCB (AUC = 92%, *p* < 0.01) and TEPS (AUC = 88%, *p* < 0.01). The details are shown in Fig. 3.

## Discussion

Vocal emotion perception is increasingly regarded as an important aspect of dysfunction in language use and social communication of patients with schizophrenia [9, 22]. This study provides a new research method for detecting vocal emotional perception in patients with schizophrenia. It focuses on performance characteristics, influencing factors, and potential neural mechanisms. By investigating specific emotions (e.g., fear, sadness, anger, disgust, surprise), including different forms of stimulation (emotional recognition and intensity), this study examined Mandarin, which adopts tone and syllable timing, and expands the research on voice recognition in schizophrenia. At the same time, using a unified software, we detected the effectiveness of our methods in vocal emotion perception and its diagnostic role in schizophrenia.

Clinically, dysprosodia or dysfunction of vocal emotion perception is a cardinal feature of schizophrenia and may also be present in other mental diseases, such as bipolar disorder [9].It is worth importantly noting that when healthy people and specific patients are compared, it could not provide a sophisticated reference in differential diagnosis. However, this study provided an exploration of this technology for emotional problems of schizophrenics through systematic comparison with cognitive and classic scales.

In line with previous research [11, 23, 24], this study confirmed that compared with healthy participants, patients with schizophrenia had impaired vocal emotion recognition and intensity discrimination. In addition, this study suggests that the overall emotion discrimination ability of schizophrenic patients is impaired, affecting not only the processing of more specific negative emotional subsets (such as anger, sadness, and fear) but also non-negative emotions (such as surprise and calmness). However, among all basic emotions examined, sadness was found to be the most difficult to detect in terms of its content and intensity. This was in line with previous studies [14], and further indicated the consistency of this research method to detect vocal emotion perception.

Critically, our study found that emotion recognition tasks are more difficult for patients than emotional intensity identification tasks. As previously reported [25, 26], a dual-language processing model was identified, suggesting that different neural networks might be involved in the explicit and implicit recognition of emotion perception. The patients in the present study showed a significant impairment in explicit vocal emotion processing, which is the recognition of emotional content. The intensity of vocal emotion identification involves implicit processing, and it appears to be relatively preserved in patients. Our research results support the hypothesis that at the behavioral level, implicit and explicit processing of vocal emotion perception in patients with schizophrenia are separated.

Compared with the control group, correlation analysis revealed that the recognition patterns of different contents showed a trend of unification and simplification in schizophrenic patients (Fig. 2). This was consistent with reported changes in brain network simplification [13, 27], which were also found to be one of determinants of social cognitive dysfunction in patients with schizophrenia. In addition, this study identified a direct correlation between vocal recognition impairment and cognition in schizophrenia and confirmed working memory involvement in these processes. Working memory is considered the core component of cognitive impairment in schizophrenia, which is related to employment status and working terms [28, 29]. The clinical relevance of working memory impairment in patients with schizophrenia is largely due to the strong correlation between working memory measurements and other cognitive impairments, such as attention, planning, and memory. Pre-attention and attention processing are important predictors of emotion recognition tasks and are related to sensory processing, especially basic auditory processing of pitch and intensity, which is significantly related to defects in vocal emotion recognition [23, 28, 29]. The present findings supported this cognitive mechanism.

In terms of clinical factors, this study did not find any significant correlation, including sex, age, disease course, and dosage of the patients. This is in line with the existing literature. Similarly, previous studies have found no differences due to intelligence, education, medication, etc. [24, 25, 30]., suggesting that it is related to the brain chemistry of the pathological mechanism of schizophrenia itself, specific brain circuits (or information processing pathways), neuroanatomical abnormalities, and environmental factors. With regard to clinical phenotypes, this study did not find a correlation between vocal recognition and the severity of schizophrenia, which was consistent with some studies [31]. Other studies, however, have suggested that the PANSS score was negatively related to language recognition and even specific positive symptoms, such as hallucinations and delusions, usually aggravate the impairment of vocal and emotional recognition in patients with schizophrenia [8, 32, 33]. More recent studies [25, 34] suggested that negative symptoms, such as loss of pleasure, might be closely related to patients’ vocal emotion recognition defects. However, this relationship was found through behavioral and neurological research techniques (including MRI or fMRI), not through clinical scale evaluations. Of note, vocal emotion perception in patients with schizophrenia is a complicated process that is influenced by various complex factors.

This study provides a good voice recognition tool based on a series of studies [22], and this clinical tool has relatively reliable sensitivity and specificity based on our ROC analysis (Fig. 3). We screened the difference between voice recognition and intensity recognition using our software, which provides a potential determination of the clinical phenotype of patients with schizophrenia in language dysfunctional processing. However, the specificity of this tool for schizophrenia needs further investigation.

Patients with schizophrenia usually perform poorly when dealing with emotional rhythms. Thus far, emotional prosody has been regarded as an important window to detect impairments in schizophrenia, and it has also played a role in improving people’s social communication. Many studies have shown that vocal emotion recognition is an important supplementary clinical index for the early detection of schizophrenia. However, this study provides a good potential clinical tool and significant evidence for the impaired relationship between vocal recognition and cognitive function. This points out an important direction for future research on emotional prosody understanding to be extended to other neurocognitive fields, such as memory, monitoring, thinking and reasoning, reading and writing ability, language production, and problem-solving ability.

### Electronic supplementary material

Below is the link to the electronic supplementary material.

Supplementary Material 1: Supplementary Methods.

## Data Availability

The datasets used and/or analysed during the current study are available from the corresponding author on reasonable request.
