# Supplementary material for: Vocal emotion perception in schizophrenia and its diagnostic significance
Source: BMC Psychiatry. 2023 Oct 17;23:760. doi: 10.1186/s12888-023-05110-2 (PMC10580536; doi:10.1186/s12888-023-05110-2)
Supplement: Supplementary file 1 — Supplementary Material 1: Supplementary Methods. [file 12888_2023_5110_MOESM1_ESM.docx]

**Supplementary Methods**

**Neuropsychological and psychopathological assessment**

**Temporary Experience of Pleasure Scale (TEPS) [1]**

Chinese version of TEPS comprises 20 items with the same response format as the original English version, i.e., using a 6-point Likert scale (from 1 = very false for me to 6 = very true for me). Temporal Experience of Pleasure Scale (TEPS) to specifically capture these two distinct constructs of anticipatory and consummatory pleasure. It is a short question- naire with 20 self-reported items (10 items for anticipatory pleasure, 8 for consummatory pleasure) allowing for easy administration.

**Personal and social performance scale (PSP) [2]**

The PSP mainly evaluates the function of patients in four aspects, and another total score was assessed according to the scoring criteria. The range of the total score is 0–100 points, which is divided into 10 grades. A total score of 71–100 points indicated that there was no difficulty or only a slight difficulty in social function and interpersonal communication; 31–70 points indicated different degrees of capability defects; and ≤ 30 points indicated that the function was low and the patient needed positive support or close monitoring. In this study, the patient’s functional outcome was evaluated using only the total score .

**Positive and Negative Symptom Scale (PANSS) and Brief Negative Symptom Scale (BNSS) [3]**

were used to evaluate psychotic symptoms in schizophrenia group. PANSS scale adopts the method of other evaluation, which includes three subscales: positive symptom subscale (7 items), negative symptom subscale (7 items) and general psychopathology subscale (16 items), with a total of 30 items.The severity of negative symptoms in schizophrenia was evaluated using the BNSS, which had 13 items, including 6 subscales: anhedonia, distress, asociality, avolition, blunted affect, and alogia. Each item was scored using a 7-point scale (0, “normal”; 1, “suspicious”; 2, “mild”; 3, “moderate”; 4, “moderate to severe”; 5, “severe”; 6, “extremely severe”). The score of each dimension is equal to the sum of the items contained, and the total score of the scale is equal to the sum of the points of each dimension. The total score of scale ranged from 0 to 78. The higher the score, the more serious the negative symptoms.

**MATRICS consensus cognitive battery (MCCB) [4]**

Neurocognition was evaluated using the Chinese version of MATRICS (Measurement and Treatment Research to Improve Cognition in Schizophrenia) Consensus Cognitive Battery (MCCB) , which includes 10 tests measuring 7 domains: speed of processing, attention, working memory, verbal memory, visual memory, reasoning and problem solving, and social cognition. The composite and subtest scores from the MCCB were converted from raw data to T-scores with a mean of 50 and a standard deviation of 10.

**Vocal emotion cognition and intensity tests**

The vocal emotion recognition task comprised 42 standardized emotional voices, performed by two professional sex-specific drama actors, including seven emotions: anger, calmness, disgust, fear, sadness, irony, and surprise. There were six voices for each emotion, including three male and three female voices. High and low materials were also manipulated, with different emotional intensities. The intensity was graded on a 100-point scale. The higher the score, the greater the emotional intensity. After completing the phonetic emotion judgment, the subjects entered the next trial, and all trials were presented in a pseudo-randomized order. The evaluation indicators included the emotional category score (the number of attempts to correctly identify a certain phonetic emotional category, with a maximum score of six points) and emotional intensity score (the corresponding emotional intensity after correctly identifying a certain emotional category, with a maximum score of 100 points).

In order to evaluate efforts of participants in the test, a repetitive voice was added to each of seven emotional voices as quality control. Therefore, it was required to complete a total of 49 voice tests. In practice, participants needed to wear headphones in a quiet room, and firstly completed six simulation tests on the computer to understand the experimental tasks. After they fully understood requirements, the formal test was conducted. After listening to the emotional voices, participants clicked the corresponding progress bar with the mouse to judge the emotional category and responded emotional intensity of the voice just played. It usually takes about 6~8min to complete one category of emotional voice tests. Vocal emotion recognition was evaluated by a standardized computer program. Quality control was added to control the analysis. Computer operators were trained before the study.

**Reference:**

1. Chan, R.C., et al., *The Temporal Experience of Pleasure Scale (TEPS): exploration and confirmation of factor structure in a healthy Chinese sample.* PLoS One, 2012. **7**(4): p. e35352.

2. Nasrallah, H., P. Morosini, and D.D. Gagnon, *Reliability, validity and ability to detect change of the Personal and Social Performance scale in patients with stable schizophrenia.* Psychiatry Res, 2008. **161**(2): p. 213-24.

3. Kirkpatrick, B., et al., *The brief negative symptom scale: psychometric properties.* Schizophr Bull, 2011. **37**(2): p. 300-5.

4. Zhang, H., et al., *Meta-analysis of cognitive function in Chinese first-episode schizophrenia: MATRICS Consensus Cognitive Battery (MCCB) profile of impairment.* Gen Psychiatr, 2019. **32**(3): p. e100043.
